# Distance caregiving using smart home technologies: balancing ethical priorities in family decision-making by only children

**DOI:** 10.1186/s12910-025-01210-8

**Published:** 2025-07-03

**Authors:** Yi Jiao Angelina Tian, Michael Dunn, Silke Schicktanz, Julian Savulescu, Tenzin Wangmo

**Affiliations:** 1 Institute for Biomedical Ethics, University of Basel, Bernoullistrasse 28, Basel, 4056 Switzerland; 2 Centre for Biomedical Ethics, Yong Loo Lin School of Medicine, National University of Singapore, Singapore, 119228 Singapore; 3 Institut für Ethik und Geschichte der Medizin, Universitätsmedizin Göttingen, Georg-August-Universität, 37073 Göttingen, Germany

**Keywords:** Transnational distance caregiving, Ethics of care, Smart home technologies, Ethical considerations in decision-making, Responsibility, Replacement of human care, Only children

## Abstract

**Background:**

The parallel growth of population ageing and international migration have introduced a unique population of transnational caregivers in elder care. Particularly for only children who face conflicting obligations and reduced caregiving resources, smart home devices could be technical tools to care for older parents from a distance. Research towards the use of these technologies has unearthed ethical issues such as privacy, autonomy, stigma and beneficence, but has not been fully explored in distance care. In this paper, we explore the ethical issues expressed by a group of only children towards integrating assistive, monitoring, and robotic technologies in their transnational care plans.

**Methods:**

Purposive snowball sampling was used for the recruitment of 26 distance caregivers aged between 28 and 45, who were their parent’s only children. They lived in Europe for at least 5 years, with at least one parent residing in the home country. In semi-structured interviews, participants discussed the ethical issues of wearable devices, ambient and visual remote monitoring technologies, as well as the possible use of one assistive robot in the context of distance caregiving for older parents. We used the applied thematic analysis methodology to analyze the data.

**Results:**

We highlight two ethical considerations. First, participants saw the need for maximizing good outcomes in caring for their older parents and fulfilling their responsibilities to ensure their health and safety, balanced against the respect for the parents’ autonomy, dignity, and privacy. Second, they weighed the benefits and harms of technologies at a distance to provide companionship and support against the intrinsic value placed on care received from one’s only child.

**Conclusions:**

Discussions to involve technologies in elder care at a distance prompted complex decision-making processes to balance, weigh, and rationalize their ethical concerns as foreseen by the caregivers. The importance of maximizing the health and safety of older parents came at an unavoidable cost of the respect to autonomy, privacy, and dignity. Participants valued their own emotional connection and relationship to their parents, which they prioritized above the instrumental value of technological support. We further discuss our findings within the ethics of care theory and concepts within transnational care literature to make sense of the broader ethical implications of this empirical study.

**Supplementary Information:**

The online version contains supplementary material available at 10.1186/s12910-025-01210-8.

## Background

### Transnational elder care

The World Health Organization estimates that persons over the age 60 years or older will continue to increase to around 22% or 2.1 billion persons by 2050 [1]. The ageing process involves a multifaceted set of growing needs in mental, physical, social and economic aspects. The consideration for appropriate care provision is required to ensure that these increased life-years are enjoyed suitable to the wishes of each older person. Much of these elder care responsibilities currently reside with family members. In the United States, around 16.8% of the national population are informal caregivers to a person aged 50 years or older, 42% of all informal caregivers provide care to their own parents (excl. in-laws) and 89% of the care recipients are related to their caregivers [2].

Alongside population ageing, international migration has also been on the rise [3]. Literature on transnational care has evolved to examine the provision of care between family members who no longer live in the same country. Baldassar and Merla [4] posit the idea of *care circulation* to capture the commitment of exchanging attention or support and fulfilling some care responsibility across distance and national borders. Although transnational caregiving is impacted by physical distance due to time zone differences, additional expenses for travel, or opacity in information exchanges, the reciprocal and asymmetrical nature of transnational care has involved creativity that maintain these acts of care at a distance. Beyond the exchange of financial support in the form of remittances, distance caregivers use alternative opportunities for *co-presence* and emotional or social support through communication technologies [5–7]. Madianou [8] contributes further with the idea of *ambient* co-presence, as well as Baldassar and Wilding’s [9] concept of *digital kinning*, which both incorporate the use of developing social networking technologies to allow a more holistic presence of the distant other in the family network.

Transnational care for older persons necessarily involves practical difficulties for sufficient hands-on caregiving (i.e. supporting with activities of daily living). Therefore, the distance caregiver^1^ often engages in a division of labor with siblings or the spouses of older persons, where the local networks of support are able to provide hands-on care [10–12]. Where a division of labor is not possible for the only child, the distance caregiving strain could be even more challenging. In an empirical interview study by Gui et al. [13] on Chinese only children living in Canada, the participants struggled between their own opportunities abroad, their commitments to care for their parents, cultural expectations for hands-on caregiving, as well as practical constraints to care transnationally. Despite being at a distance, the participants had reservations about offloading their elder care responsibilities to formal caregivers or admitting their parents to nursing homes. Under these conflicting considerations, only children^2^ living aboard with caregiving responsibilities to older parents face a niche set of challenges that require attention into the tools that could allow the older persons’ needs to be sufficiently met.

### Conducting care activities using smart home technologies

The care ethics literature offer conceptual and normative theoretical resources that have not only been influential in ethics scholarship, but also hold thematic relevance to the topic of care and familial relationships. One dominant contribution was provided by Fisher and Tronto, who highlighted the notion of care as a *species activity* that is inherently relational and shared amongst all individuals, as a social effort essential to improving our collective well-being [14]. They distinguished the multi-faceted nature of care activities that is carried out in four intertwining phases: (1) *Caring about*: the act of paying attention to and recognizing care needs; (2) *Taking care of*: having responsibility to handle and resolve care needs; (3) *Caregiving*: having the competence to resolve care needs with action; and (4) *Care receiving*, the active reaction from recipient to care given. These care activities may present itself in varying intensities; involve different tasks and responsibilities, with different competencies required and from different roles in care.

Recent technological advances have also enabled the possibility to monitor the vital signs, movements, and the safety of older persons in their home environment, while providing real-time information to caregivers remotely [15–18]. Wearable technologies could measure physiological conditions such as heart rate, activity level, blood oxygen levels, and sleep; ambient sensors at home could detect environmental risks, signs of wandering behaviors and memory loss, or falls; and visual sensors enabled by AI recognition could detect and even predict falls and abnormal postures with greater accuracy. Robotic technologies for service tasks and companionship may also aid elder care [19, 20]. Such new technologies could potentially provide distance caregivers with more creative opportunities to contribute to the care of their parents despite being at a distance.

The implementation of technologies in the care of older persons has been scrutinized for ethical concerns [21–24] such as privacy, autonomy, dignity, trust, justice, reduction of human contact, and stigma. Issues of autonomy come into play when considering whether older persons have consented to the use of technologies in their homes or care [25]. After the technologies are implemented, any discomfort of feeling surveilled or watched in the home environment would be relevant to both concerns for privacy or dignity [26, 27]. Subsequently, the constant collection of potentially private and sensitive information about one’s location, vital signs, medical visits, and visual information of oneself and one’s home environment warrants considerations of data protection and privacy [26, 28]. The issues of social isolation and social care could be relevant if the caregivers reduce their in-person visits after receiving sufficient information of their parents’ health statues from remote monitoring technologies, or if the companionship from robotic technologies provides novel opportunities for social contact for older persons [29].

### Aims of the present paper

Smart home health technologies could be a promising tool for elder care, but existing research reveal many ethical concerns that require careful balancing and considerations to the interest of all those affected by their use. To our knowledge, no empirical investigation and analysis of the distinctive ethical challenges facing the adult only child making use of remote monitoring and robotic technologies to supplement their care to older parents across national borders have been conducted. As an empirical-informed normative paper, we also draw upon the ethics of care theory proposed by Fisher and Tronto to make sense of and discuss the implications of the empirical claims made by the participants as contextualized by the challenges in transnational care.

Our research questions are as follows:

1. What ethical issues are raised by participants when considering to integrate assistive, monitoring, and robotic technologies in their distance caregiving plans?
2. How would participants reconcile ethical concerns with the opportunities that the technologies could provide to alleviate their distance care challenges?

In investigating these questions, our paper sheds light into the ethical considerations of a unique and unexplored demographic of only child transnational caregivers. Our findings may be beneficial to inform the design of technologies and drafting of policies that may allow this population of family caregivers to best make use of caregiving tools for the care of their parents.

## Methods

### Ethics approval

The Ethics Commission of Northwest and Central Switzerland approved the study, ID: AO_2020-00027. An information document and a consent form detailing the background and aims of the project, procedures taken for data protection, as well as the possibilities to withdrawal were provided to the participants before an interview was scheduled. After the participants read both documents and their questions were answered, a suitable time for the interview was scheduled at their convenience. Prior to beginning the interview and recording, the consent form was verbally reviewed together with the participant and any further questions were answered.

### Study design

We employed an explorative qualitative methodology using semi-structured interviews to study the responsibilities, challenges, and caregiving arrangements of only children living transnationally from their parents. The study participants were recruited using purposive and snowball sampling. A three-part interview guide was used: (1) Participant’s current caregiving arrangements, thoughts about their responsibilities at a distance, and challenges to providing care; (2) Perceptions of opportunities, areas of improvement, and ethical issues of using remote monitoring and robotic technologies in their own distance care; (3) Broader societal impact of using technical innovations in elder care and their own relationships with their parents. This paper originates from a larger project on the use of smart home health technologies for the transnational care of older parents. The interview guide may be found as a supplementary material 2.

### Study participants

Eligible participants were: (1) An only child with no siblings, with sole responsibilities for their parents (not including in-laws); (2) Living and have lived in Europe (including UK) for more than five years prior to the interview without short-term plans to repatriate to their home country; (3) With parent(s) residing in the home country (excluding Europe, to account for the heightened bureaucratic and political barriers for family reunification from those living outside of the EU, as travel within the EU and for EU citizens exist greater freedom of movement) and do not require immediate or long-term hospitalization; (4) Between the ages of 30–50, or not still in post-secondary education as an international student (if under 30) and do not need care themselves. Recruitment strategies include the distribution of a flyer with the eligibility criteria and the aims of the study to local migrant and expat organizations, social media platforms, word-of-mouth, and through university-networks around Europe. A total of 25 interviews with 26 participants (Table 1) were conducted between May 2022 to March 2023 and recruitment stopped when data saturation was reached. As data collection took place during the fluctuating restrictions of COVID-19 and changing health recommendations for distancing and masking measures, all participants were provided the option to partake either in-person or over a video-conferencing software (Zoom). To allow interviewees to express their thoughts comfortably and in a language that they are most familiar with, the bilingual interviewer offered to conduct the interviews in either English or Mandarin Chinese. All interviews but one was conducted over Zoom, with twelve split into two sessions. The average length of the interviews were 127 min, spanning a range of 77–176 min.

**Table 1:** Participant demographics* (*N* = 26)

	Demographic ratios
*Gender*	Women: 12| Men: 14
*Highest education level*	Ph.D.: 9| Master’s: 10| Bachelor’s degrees and lower: 7
*Age group*	20–30 years: 1| 31–40 years: 16| 40–50 years: 9
*Place of residence*	Germany: 18| Switzerland: 4| Other European Countries: 4
*Parent’s location*	China: 22| South Korea: 1| US: 1| Lebanon: 1| Turkey: 1
*Family statuses*	Unmarried: 6| Married (with children): 20

*one interview with an only child and his spouse (also an only child)

### Data analysis

All interviews were transcribed verbatim in English and those conducted in Mandarin Chinese were translated and transcribed by the bilingual interviewer YJT. The method of applied thematic analysis was used, as we strived to present the collected data in a manner that is transparent, accurate, and comprehensive [30]. This method allowed us to inductively identify the ethical concerns raised by the participants in the context of their experiences in transnational care, rather than to validate a certain theory or epistemology. The initial rounds of data familiarity and coding was completed by YJT and TW in order to seek coherence in the interpretation of the data and to build the foundations of a coding tree for this paper. The overall direction of and the broad inclusion of the data for this paper were determined together with SS. A selection of codes are provided in the supplemental files as an example of the process. Reiterative rounds of applied thematic analysis were then conducted between YJT and MD to determine the overall structure and improve the coherency of the findings, including the final confirmation of the two clusters of considerations and the selection of quotes. Subsequent and repetitive rounds of reflection to establish the significance of our findings in the context of the larger body of literature were first conducted between YJT and JS, then with all co-authors both individually and collectively in order to confirm the final structure of the paper.

Our study was designed to elicit the participants’ attitudes and ethical concerns towards the use of technologies in their parents’ care. During the analysis phase, we noticed that the participants’ ethical concerns sharpened and converged largely in the context of decision-making in their anticipated care plans to integrate technologies, where they tried to balance and weigh varying considerations against each other. The findings presented below was the result of our analysis to reflect the processes of balancing, weighing, rationalization, and prioritization of these ethical concerns.

## Results

The following section presents the ethical concerns raised by participants when they are prompted to consider to integrate technologies in transnational care. We identify two overarching considerations that involve weighing, balancing, rationalizing and operationalizing these ethical concerns. The first consideration involves participants prioritizing the best outcomes in care through technology use, constrained against the principles of autonomy, privacy, and dignity. The second consideration in their decision-making involves participants recognizing the unnaturalness and unacceptability of replacing human caregiving by technological tools, balanced against their need to meet the unique demands of distance care and reduced social contact from family.

## Consideration 1: Maximizing good outcomes in care whilst balancing ethical constraints

As the only children and often the primary caregivers, participants evaluated a technology’s potential to promote the physical well-being and safety of their older parents. Nevertheless, participants were also aware of the invasiveness of these devices and potentially negative reactions from their parents to allow remote monitoring of their own homes. In this section, we present a consequentialist operationalization of prioritizing care responsibility to maximize parents’ physical well-being as the main orientation for the participants’ motivation to use smart home technologies in the distance caregiving for their parents. We also examine the ethical concerns of autonomy, dignity, and privacy as constraining this orientation.

### Prioritizing care responsibility to maximize parents’ physical well-being

For distance caregivers who faced physical and temporal barriers to receive adequate and timely information from their parents, equipping their parents with either wearables or remote monitoring sensors in their homes could allow any health deviations to be detected and appropriately attended to. For the wearable technology that could monitor for the heart rate, activity levels, basic cardiovascular function, as well as the detection of falls, participants saw benefits for chronic disease management and promotion of physical activity. Ambient and visual sensors could allow the prediction of falls and the prevention of maltreatment from formal caregivers, as the only children at a distance are not expected to be capable of providing bedside support.

Participant 12: I’m very happy that both of them continue to wear their Apple watches, because the Apple watch is…it has that functionality, […] it has the sudden fall technology built into it. So if there is a fall, the phone says, do you need me to call SOS, and if you don’t respond in a certain amount of time, it automatically calls, and both of their watches have that functionality. I feel good about that already, you know?

Participant 23: In the bathtub, to have such a monitoring device is necessary. If…like what you said, there is one installed on the floor, once the older people have slipped, or they don’t get out from the bathtub for a long time, then that is a problem. Like it is a warning, or some information, this is pretty great.

Gaining access to their parents’ health data collected from the technologies was seen as justifiable as long as the familial caregiver has the best interest of the older parent in mind and could use the data to promote their abilities to fulfill their caregiving duties. In this way, even if distance caregivers could not necessarily offer concrete support, there would still be many benefits from staying updated during an ongoing medical event or to participate in the communication with physicians.

Participant 18: Like…at a distance…it’s like, just an example, if they were to press it and then…the ambulance has not come in a bit of time, but I’ve received it here, then I could go and immediately contact the people I know and are friends with, to see whether someone is nearby to take a look. To go and help a bit first. Because I don’t know what their specific problem is, but at least when I am at a distance, “Oh their heart rate is very slow or their blood pressure is very high, ” then I could…when I am calling, I could say that “Their blood pressure is very high or their heart rate is very slow.”

In moments of high dependency, health deterioration or imminent risk, a couple of participants also expressed the view that it would be justifiable to prioritize the benefits brought forth by technologies and install them despite and against their parents’ resistance. One participant rationalized his decision by postponing the conversation with his mother and first installing the sensors, as it would be more important to ensure that everything was done to ensure her physical safety and well-being before anything else was considered. Another line of thought also tied to high dependency, was the debate about whether it was possible for those who have lost their cognitive abilities to consent, in which case the monitoring technologies would also become more necessary.

Participant 22: There is only one scenario [to secretly install ambient sensors], for example if they… if they are severely sick and I have to do this, but I don’t want to hurt her ego, I may choose to do this. […] If she really had signs of dementia, for example, under this situation, then of course I have to…of course…when should I tell her? If I haven’t made a decision then I could first consider using such technology and wait until a good time to tell her. Like…that’s the situation.

Participant 15: I think maybe it’s like…from the perspective of considering their health, that it is necessary to use this camera to monitor their actions, but…[when] they still have their ability to judge. So firstly, their brain must be conscious. If they were lying in bed, then you don’t need to negotiate with them. If their brains are conscious, with their personality, they would definitely be against this matter. [If] I still think that this is definitely necessary, then I would install it without them knowing.

Similarly, as participants are making caregiving decisions within the family and relatives, some rationalized the prioritization of well-being based on a prior knowledge of their parents’ personalities and long-standing wishes. Two participants were confident that parents would be fully aware and in align with his interests to promote the maximization of good outcomes in care, and there would be therefore no need to install without consent or overriding any unwillingness to accept technologies. For one, a verbal rejection from the parents may not truly reflect their wishes, as this rejection may arise more from the fear of bothering their children.

Participant 5: I think my parents would…would probably also agree to it. They probably won’t…won’t have an objection.

Participant 9: So just to tell them that these have benefits for the body, take a look, but actually they are passively wearing this monitoring device, I won’t tell them. Because…like what my wife said, at the end they….so parents wish that you are by their side, but Chinese parents wouldn’t want to increase too much of your burden. They are even very conflictive [about] these things.

Extending this to the type of data shared, some participants believed that there should not be many restrictions even with the sharing of sensitive visual data as they were part of the immediate family.

Participant 24: I think this…could be the purpose of this product to protect privacy, but I think if it is just between relatives, it is not necessary.

### Constraint 1: Respect for autonomy

Despite the valued benefits to ensure safety and receive timely alerts on the statuses of older parents, some participants expressed discomfort at the invasiveness of the technologies. Where they anticipated negative reactions from the parents, most emphasized the importance of respecting parents’ expressed wishes. The home was seen and valued as a place of comfort and freedom from restraints, where older parent should be able to act autonomously in line with their own routines and habits.

Participant 7: So I just want to say, if this was shared…it would be like being in a boarding school, all controlled. I think there doesn’t necessarily need to be that much control and monitoring.

After weighing their orientation of maximizing good outcomes in care with the use of technologies, some participants expressed conflicts when balancing against the respect for their parents’ autonomy where they anticipated negative reactions from parents. Many participants thought that the respect for the autonomous wishes of the person should be prioritized above any other considerations like their health or providing reassurance for the caregiver.

Participant 16: Feelings? The first impression is that it is a human right, the right of an individual, the individual right. Perhaps…my mom would want to wear it today, but not tomorrow. That is…right? It depends on her own mood…it is something that you could and also don’t have to wear. […] But I hope that they wear it. (laughs)

In cases where the participant anticipates clear refusal to use the technology from a parent, many participants thought the secret installation and use of the technology was disrespectful to their parents’ autonomy, even though it would be beneficial for their care.

Participant 3: No no, they definitely need to know whether there are cameras. Right. You can’t install a camera and they don’t know. When they are monitored without knowledge, I think that is even more unacceptable.

Participant 26: Hmm, I wouldn’t, I would just be very transparent about what it does […] and turn on the…whatever feature that is, and just measuring your…your…biometric information and yeah. I’m gonna respect their choice.

Responding to the tension between maximizing good outcomes for the parent’s well-being and respecting their autonomous wishes that refuse the technologies, participants re-orientate their goals in ways that operationalized a balance between these competing ethical values. Some participants suggested approaches that they thought allowed the benefits of the technology to be realized whilst simultaneously acting to enable their parents to maintain some control over its use.

Participant 6: So with the camera on, you have less privacy. So maybe I…if there is a choice of turning it off by myself, then I think that it’s fine. And also robot as well, so it definitely has a camera too. So but given that the choice is there about turning on or turning off, then…I think it’s fine. I wouldn’t mind it, yeah.

The non-ideal realities of caregiving at a distance also motivated operational decisions to use technology to ensure good outcomes for parents as compared to reasonable alternatives. The justification for using technology was seen as the “less invasive and more autonomous option” when compared to, for example, the hiring of a human caregiver or a move to a nursing home. For example, if parents were uncomfortable with (or disliked) a paid caregiver, one participant suggested that it would be easier to turn a device off than to fire a caregiver. She also questioned whether the standard procedure of hiring formal caregivers would also be otherwise discussed thoroughly and consent fully taken with the parents, whether it would be ultimately even more limiting for parents to have a dissatisfactory caregiver that is more challenging to part ways with.

Participant 1: If they could live alone and have the help of these sensors, so I think maybe this is better than if they lived with a paid carer […] Maybe a lot of children now that would pay for caregivers, or to provide these…is it really discussed thoroughly with the parents? Could the parents fire these caregivers? This is what I think needs to be thought about.

### Constraint #2: Respect for dignity

Particularly with the use of cameras, a few participants made associations with the monitoring of infants, pets or prisoners, which they recognized as disrespectful and undignified towards their parents. This concern went beyond simply failing to act in line with their parents’ autonomy, as they should not be monitored in this way even if they are incapable of consent or even with high-dependency and necessity to use the technologies. For some participants with an Asian background, there was also the sense of importance attached to respecting their parents and were particularly uncomfortable with the idea of monitoring their parents, somehow as lesser-than-equals with them.

Participant 22: In whatever condition my mom may be, I would not want to use this thing to surveil her. Isn’t it just a prison?

Participant 21: If I have installed this thing and I don’t tell them, then I think…this is a very disrespectful behavior. Because no matter what age someone is, they still…have their own will. They are not some puppet that could be controlled at any time by someone.

Although they emphasized the importance of doing what is best to maintain their parents’ health and well-being, it became clear that participants had certain conceptions of what may allow them to have a comfortable life that constrained them from accepting certain technologies in care. If one were to be monitored and controlled to live a restricted life, only to be completely healthy, this was not seen as conducive to living a joyful life. Furthermore, older parents who may naturally be strong-minded and independent, introducing the technologies could make them feel weak and dependent on others.

Participant 20: If they are protected but it has made them feel that their life is very…they are constantly living under like on the boundary of death, like in a state of fear. […] Then I would rather for them to live more fulfilled every day, and not to remind themselves every day that “I am now old and I am almost done.”

### Constraint 3: Protection of privacy

An efficient and rigorous protection scheme against the misuse of data, such as for purposes that were not prior agreed to or those that may be used to harm the older person, was expressed as the main constraint that followed from a concern about privacy. Participants expressed ambiguous views about whether data should be shared with distance caregivers. While some participants would value the information and reassurance from the device, others believe that distance caregivers’ limited capabilities to provide immediate support may not be proportional to the invasion to their parents’ informational privacy. For the following participant, informational privacy acted as a constraint to his responsibilities as a familial caregiver. He associated the access to data as having the tool to provide efficient caregiving, where the distance caregiver should not be a prime candidate as they are neither a medical professional nor located in the immediate vicinity to provide timely support:

Participant 2: This is also a problem, because actually a lot parents, they would also want their own privacy, their own private sphere. Like, you would even want to see my heart rate? You are my child, not my doctor.

In contrast, socio-cultural understandings of family and responsibilities led to a different balance being struck between the need to access personal data and the requirement to keep these data confidential:

Participant 19: I think, traditionally…from a Chinese…or Asian culture, there should not be any privacy between children and their parents, it is completely transparent, whatever you are doing parents should always know. But I think there should also be privacy between parents and children. And this…(unintelligible) also falls under the jurisdiction of privacy.

Possible solutions to maintain privacy were viewed negatively. Most participants dismissed the option to manipulate the camera to blur out the parents’ faces or to only show a silhouette image as futile, as it would be obvious to the children that they are the parents. A couple of participants suggested that adopting this approach would not only feel strange, but could put more distance between the distance care relationship, “as if you are watching someone you did not know.”

Participant 19: Then I would feel even worse. Right? I know exactly this person and you still deliberately conceal…like…it is as if this feeling was originally that you are observing a third-party….or someone or something who is being observed, and you go and deliberately blur it out. The result from blurring it is actually expanding this distance even more. Right? So in my perspective, this is even more…even more…I cannot put it in words, so just a very strange type of feeling. (laughs)

Operationally, a more conducive balance between promoting good outcomes and respecting privacy was seen by devising technological approaches in which a clear delineation of temporal, physical, and caregiving roles was established:

Participant 4: It doesn’t have to be made so complex, it could be okay if there are monitoring just in the high-risk areas. For example, bathtubs…falls, I also heard that accidents often happen there. So maybe have sensors in these areas. However, whether the TV is turned on or whether the doors are open, I think for the people being cared for, they may not want their every action to be seen, or something.

In this first consideration, participants expressed their commitments to keep their parents safe and healthy through the studied technologies at a distance. Nevertheless, they were also concerned about the devices’ invasion of their parents’ rights to autonomy, privacy, and dignity if technologies were implemented against their wishes, without their knowledge, or overrode a sense of respect to their parents. These concerns were organized in such a way to realistically present the complexity of the entanglement and interplay of these ethical issues during the participants’ contemplations during data collection, rather than in isolation and as distinct principles.

## Consideration 2: Ensuring human companionship whilst addressing reduced social contact

The reduction in direct human contact was identified as one of the most significant ethical concerns in the distance caregiving context. Due to time differences, financial and temporal constraints for frequent physical visits, and the conflicting obligations to their own nuclear families in the host country, participants often expressed regret for being unable to physically provide company to their parents and contact them as much as would be desirable. This second section engages with the main orientation of the intrinsic need for familial human care as balanced with concerns of replacing the reduced social contact in distance caregiving through the use of companion robots. Issues relating to stigma, cultural concepts of familial relationships, and infantilization are also discussed.

### Intrinsic need for familial companionship

In general, participants thought that nothing would be presently capable or ideally should replace the inherent preference to converse and bond with one’s only children through human caregiving activities. Technology was merely an assistive method. For the participants, there was something intrinsically desirable and irreplaceable about the physical presence of a loved one.

Participant 12: Ahm…well, the first thing that comes to mind is, just like, there’s something about…the physical presence of a loved one, that would…you just can’t substitute, you know, this is, we talked about last time, like, if something were to happen, I’d be there tomorrow…to uh, to hold hands. And that, that’s something that no technology can substitute.

In the same manner, participants felt that a child or a spouse would be irreplaceable, even by the best technology or formal caregiver, simply for being who they are and their role in the caregiving relationship.

Participant 22: Even if you hire the best…formal caregivers who have received the best education to provide services, they still may not be satisfied. Why? Because they are not their biological children. That’s how one could put it.

A few participants worried that gifting or introducing the idea of the robotic technology could provide the wrong message to their parents, that this would signify a withdrawal of attention and care from the children themselves. One participant also worried that conversing with the robot would only exacerbate the forlorn situation of their physically absent children.

Participant 13: Hm. I mean obviously they, they would probably find it very weird (laughs) if I…show up with that little guy. You know, it sends a signal, obviously. It sends a signal, because you are trying to replace the caregiving…that I, as their son want to give them. I mean, it’s more like a substitution. […] Yeah “here you go, I don’t want to care about you anymore, here is a robot, that’s it.”

Whilst participants found the emotional care and companionship that they provide to be intrinsically valuable and irreplaceable, they were also aware that they would be unable to provide the same level or quality of social care, entertainment, or companionship at a distance. Three distinctive types of responses were identified as participants attempted to appropriately position the use of technologies against their prioritization of the value of human caregiving.

### Response 1: Supplementing emotional connections

Not all participants were against their parents forming attachments to a robot, or for its presence to compensate for the children’s physical absence, particularly for those whose parents were living alone. Participants felt that some emotional connection could be provided in the form of telling stories, interacting or conversing with older persons, or by simply being present that allow older persons to feel less alone.

Participant 2: They only need someone to tell them stories, because they would feel very bored. As someone to talk to. When they are both alive, then this would not be a problem. The problem comes when one of them dies, when the older person is alone. This is when they would need someone to talk to, to have conversations with. This is how we may help them.

Participant 13: I think it would provide some kind of…some kind of possibilities for interaction? You know? As I said, it will not replace the human part, but I mean if you imagine they’ve been married for, I don’t know, half their lives, or even more…and when obviously…the other part dies, I think…it could just be empty. This could probably maybe help a little bit, a little bit.

In a similar vein, the use of non-robotic or non-humanoid technologies was preferred as it would not compromise the human connection because there is a visible difference from human caregivers. Rather, a non-humanoid robot would provide the emotional benefits and convey care, but would be easier for older persons to accept.

Participant 20: I am very sensitive to the things relevant to appearance. I would feel like it really looks too much like a robot, it would make older people have a feeling like…there really isn’t anybody there to care for them, there only is something that emits electronic sounds. It feels like there is a stupid…like a fake human to care for them. So I would feel like…if there was small furry…something that could make them feel, on the one hand make them feel better inside, to let them….as a way to help them alleviate.

### Response 2: Increasing health needs call for more support

Due to the financial capabilities of this sample of distance caregivers and the lack of resources from having other siblings to share the physical caregiving tasks, participants were open to and planned on hiring paid caregivers (or a move to nursing homes) if parents became more dependent. Therefore, having technologies to provide information or potentially assist in tasks of daily living were often compared with the capabilities of formal caregiving. When nevertheless stepping back to consider the overall shortage of human personnel in nursing homes, and the limitations of live-in caregivers who are not family members, participants looked positively towards the future capabilities of technologies to supplement or replace the meeting more of their parents’ health needs (rather than only their emotional needs):

Participant 14: Now comparing it to a caregiver, of course, if it’s the way it is today, it doesn’t replace it. But if it evolves properly and there’s proper financing in it, then I think you can get to a stage where…it can substitute the caregiver and the advantages compared to a human being is that, it’s somehow anonymous. So it’s not someone who’s in your house, and intruding. It’s…a bit more…yeah, docile.

Participant 20: Hmm, if they were actually having issues with mobility, maybe if in everyday caregiving, where there are a lot of details, even if we did hire a professional caregiver, they would still be 24 h around the clock very detailed care. So if for machines, if its level of precis[ion] is very high, it could fulfill purposes of monitoring.

For more intimate tasks such as showering, toileting, and changing clothes, a robot that did not resemble a human being would be more comfortable and dignity-preserving than human caregivers. This was a promising point for the participants towards the replacement of more tasks by the paid caregiver, some even to improve the care and living experience for their parents.

Participant 12: And if it’s a robotic shower, that’s why I got excited, it’s a robotic shower, it looks nothing like a human, my parents would never think that it’s another human bathing them, right? But if it’s a humanoid robot, I think there might still be a bit of that hesitation from my parents, like the “Ughhh (repugnance), got a human to do this!”

Participant 5: It is just like a household appliance, it’s just like if you bought a TV or an AC, it’s just like a household appliance.

### Response 3: No technology should be involved in caregiving

Many participants were uncomfortable with imagining humanoid robots playing a role in caregiving for their parents, despite the awareness that their parents may have reduced social contact due to living alone or having their only children at a distance. A number of participants expressed the unnaturalness of communicating with a robot, as it is simply not possible to show that they “understand you” and have the capabilities necessary for interpersonal interactions. One participant also shared a description of the “coldness” of robots as compared to the “warmness” from biological beings that would especially be preferred in later ages:

Participant 21: Then you know, for older people, a very cold and icy robot to converse with them, would they be very happy?

Having a humanoid robot was also seen as having stigmatizing effects, on the grounds that one would be labelled by others as being “lonely” or having social anxiety:

Participant 1: Also to everyone, whether it is something that everyone wants to accept, so not just my parents and myself, but also the society, everyone’s acceptance towards it. For example, this robot…as an example in our building complex, if only my parents are taking walks with a robot (laughs) and whether the neighbors could accept it.

Participant 18: The people who would need this kind of thing are those who are very lonely, the people who have social anxiety or social phobia, those people are suitable for these….

Those participants who were uncomfortable with any use of robotic technologies in caregiving saw paid human caregivers as the only route to fulfill their parents’ care needs, and no-tech tools to resolve concerns about loneliness or social isolation. One participant was open to pinning some emotional care on a paid caregiver to balance their discomfort with using technologies to replace their own presence, but also not to rob their parents of an opportunity for social interactions:

Participant 15: Ohhh…I…think, I would want a human to take care of them, it is to make up the regret that I cannot care of them. And not just the regret, but a kind of…to lessen my burden or to alleviate some of my worries. I would pin some of my emotions on that person, on the service to my parents, and would imagine that their services to my parents, it is actually me doing the service for them. I think this is easier, in terms of shifting the emotions. And if it is a robot, I really cannot compare its capacity for work with my capacity for work.

Other participants thought that their parents would be better off interacting with human beings in their social communities or larger family networks.

Participant 9: Hmm….for my parents, I wouldn’t, because they would rather drink tea, play chess…and socialize with other older people, more of a social purpose, older people are afraid of loneliness.

The second consideration collectively presents the issues of the reduction or replacement of human care, concerns for technologies inducing stigma and infantilization, as well as prompted distinctions of instrumental and emotional care primarily relevant to robotic technologies. Devices were not deemed capable to replace care from the participants, as their parents’ only children, which prompted ethical claims about the value of human-dominated care. Nevertheless, participants suggested appropriate compromises to the technologies that made them more acceptable to be integrated in the transnational care plans, such as through resolving instrumental care needs or the devices having a non-humanoid appearance.

Overall, participants recognized the opportunities these technologies posed to increase the benefits of caregiving, but also expressed ethical concerns associated with their use. In particular, these related to concerns around privacy, autonomy, dignity, beneficence, and the need for human connection when technologies of these kinds are deployed. The central place that these concerns played in constraining the putative benefits of these technologies in participants’ accounts call for nuanced judgments in deciding about the appropriateness of specific technological interventions.

## Discussion

Our study explored only child distance caregivers’ accounts of balancing and prioritizing ethical values when deciding to employ remote monitoring and robotic technologies to care for their parents. This paper examines a unique sample of immigrants adult children on their transnational care, who are the sole children of their older parents living in the home country. In other studies discussing the use of remote monitoring and robotic technologies, rationales and ethical considerations have been interrogated in the context of having the caregiver living within surroundings of the same city from or within the same country as the older person [31, 32]. In that context, the ethical arguments developed have justified a narrower scope of technological use only to supplement care during brief-absences of or in conjunction with the caregiver. By contrast, the study participants needed to negotiate their limited resources and capabilities when tasked with providing care at a distance, unable to shoulder much of their in-person and hands-on caregiving responsibilities recognising that there were both significant benefits but also more extensive challenges associated with implementing technological tools to supplement and bridge gaps in care [13]. In so doing, our novel contribution lies in the discussion on the prioritization of considerations in the use of new technologies within family caregiving settings, shedding further light on the complexity of decisions in configurating caregiving practices at a distance.

### Balancing competing ethical considerations

In order to decide whether to use these technologies, one may be faced with the need to balance and weigh the ethical values at play [33]. Our study has revealed how distance caregivers are motivated to use technologies to fulfill their responsibilities and maintain the health and safety of their parents, but that decisions about how these technologies should be deployed involved balancing competing ethical considerations. Empirical research on the willingness to adopt technologies also noted privacy being weighed against values such as autonomy and safety [32, 34]. Wang et al. [35]’s recent conceptual review of literature found that whilst privacy concerns were raised by participants, they were still strongly motivated to adopt smart homes to increase personal safety and security in the home. Similar findings were reported by Dermody et al. [36], Wild et al. [37], and Jaschinski et al. [38]. Our study likewise revealed that, while only child caregivers were focused on fulfilling their caregiving responsibilities in improving the older parents’ health and safety as a central focus, to which other ethical concerns were weighed against. For these caregivers, recognising and balancing against their concerns to enable the benefits of the technologies was presented as an essential part of making good decisions about the use of technology, and in configuring their precise role in providing and stewarding care at a distance. This point also corroborates an existing study by Shelton, Orsulic-Jeras, Whitelatch, and Szabo [39], where family caregivers with responsibilities to older parents place more emphasis on improving their health and safety, rather than the older persons’ own preferences to maintain autonomy and other values. In our study, some participants expressed that it would be justified to act against or persuade their parents should the technologies’ benefits outweigh other concerns. This finding chimes with a previous study by Berridge and Wetle [32]’s on older mother-adult daughter dyads, where adult children expected some resistance but were confident that they could successfully persuade their mothers to accept sensors technologies for caregiving despite their anticipated refusals. The authors also warned against the underestimation of their parents’ capabilities to understand the technologies and consequent non-cooperation in seeking informed consent from their parents. However, not all of the participants we interviewed found it justifiable to simply override their parents’ wishes, expressing the view that attenuating the deployment of the technologies to their parents’ views was part of respecting their autonomy, despite the potential benefit of reassurance to the caregiver and the increased safety to the older person.

Next, the participants we interviewed prioritized the care and companionship that they would provide to their parents above any considerations of technologies or other caregiving resources. As most were able to afford paid caregivers or technologies to supplement the practice of care, the point of reference for participants’ ethical assessments of smart home technologies was predominantly compared with these alternatives to fill the gaps of care. Participants are also concerned about the technology resembling an alternative that could replace their own presence and interfere with the familial relationship they had with their parents. When evaluated within the ethics of care theory, this concern highlighted the importance of mutual dependence between the distance carer and their parents that is central to care, which should remain uninterrupted by technologies. While physical care may be offloaded to paid carers, the emotional bond was seen as integral to their relationship as a family. Nevertheless, where the use of technologies do not interfere with the replacement of care *provided by the children*, such as those hands-on tasks normally offloaded to a paid caregiver, the participants seemed to be much more open to using them in care. Likewise for robotic technologies, participants thought that some basic emotional care could be provided to older persons that would not necessarily run into the problem of replacing human care. For example, as the participants were not physically able to provide companionship to their parents, robotic companion could reduce loneliness and be a source of entertainment which do not necessarily need the intrusion of another person into the home.

In cases where practical difficulties arise to hire a suitable formal caregiver and or physically be there to take care of the parents, having technologies could be a less invasive and more autonomous option, also more comfortable for the parents, than living with paid caregivers or with many other nursing home residents. Having remote monitoring devices or a robotic companion would be much easier to simply turn off or disengage, which could also ascertain that older persons’ autonomy would be better enabled. With such claims, the participants are actively anticipating the changing needs of their older parents at the reception of technologies. Therefore, a design recommendation from our findings would be to allow customization and introduction of more user-control for the older persons into the design of the technologies were ways to maintain their benefits in care, while mitigating their ethical concerns [40]. Aligning with the ethics of care approach, the older care recipient may thus be acknowledged as an active agent with greater agency and enactment of preferences in their own care [15]. Likewise for the enthusiasm expressed by one participant towards a robotic shower for intimate care tasks, to allow for greater consideration towards the care recipient’s needs for dignity when they should be expressed by his parents, particularly inmidst of changing health needs and preferences [41].

### Technology-supported care activities at a distance

The inclusivity of four phases of care allow the technology-supported distance care tasks carried out and planned by our participants to be *recognized* under the notion of care [14]. Transnational care scholars validate this idea in the concept of *care circulation* and deem the management of care at a distance to be intensive in its own right [42]. The term *personal care by proxy* encompasses the delegation and coordination of support from the surrounding social network, communication and provision of knowledge, finances, and time that is necessary to maintain the proper functioning of a transnational care arrangement [43]. Technologies may serve in these elder care contexts as a supplemental tool to identify care needs, assessing urgency and priorities, as well as providing reassurance and assurance for care recipients [18]. In this way, care activities that involve attending to and taking responsibility for the health needs of older persons could be affected and aided by some function of technologies. When contextualized in challenges of distance care, the phases of *caring about* and *caring for* may be facilitated by the studied technologies through information provision about the older parent’s needs and the efficient marshalling of care resources, such as enabling carers to contact surrounding social networks or participating virtually during medical emergencies. Nevertheless, the phase of *caregiving* would, however, require greater physical competencies (mostly hands-on) and material support that is not seen as yet possible through technology-enabled transnational care.

### Limitations

This study has limitations. The sample we recruited inevitably affected our findings. Firstly, the participants were by-in-large highly educated and were from middle-class families in their home country, which may be related to the accessibility and openness to discussing smart home technologies in elder care. It is critical to note that we were only able to hear the perspectives from the only child distance caregivers, rather than the parents who may be most impacted by the use of technologies in their care, thus we do not know how they may respond to the accounts of decision-making expressed by their children. This may also translate to the weight of certain ethical issues presented in this paper, such as the emphasis of the feeling of stigma, which may be more strongly felt by the older parents as the end-users. As the parents were reported to be still physically and cognitive independent, without requiring substantial long-term care either in the nursing homes or provided by a paid caregiver, most adult child participants did not yet have extensive knowledge or experiences on caregiving responsibilities for more dependent older parents and what this may require. This may have influenced the participants’ attitude and especially urgency towards using technologies in their care. Our sample also primarily consisted of only children with a Chinese background between the ages of 31–40, which corresponded to the implementation of the one-child policy in China from 1980 to 2015, as well as the linguistic capabilities of the interviewer. As we also recruited using the snowball method, most participants were also living in Germany. These may have inevitably influenced the diversity of responses of our findings in regards to their cultural values on responsibility, care for their parents, and overcoming specific challenges across these specific geopolitical distance barriers. We acknowledge that participants’ cultural backgrounds as well as that of the researchers also influence the interpretation of ethical issues [44], not to mention any shifts in the conceptualization of these issues in their process of transnational migration depending on the duration, level of exposure, and acceptance of the host country’s cultural values. Due to the nature of the qualitative research methods as well as the sampling strategies, our findings are not generalizable or representative in weight or exhaustiveness of the distance caregiving population. The themes and data are only a reflection of some of the ethical considerations in deciding to use smart home technologies in distance caregiving, as presented by our participants and to the best of our interpreted analyses.

## Conclusion

Our paper contributes a unique and unexplored demographic of study, providing new perspectives on, and offering provisional ethical rationales for, the appropriate use of technologies in transnational family caregiving. The value of safety and well-being in distance care was central to family caregivers, but was seen as coming at an unavoidable cost to other ethical considerations such as privacy, dignity, and autonomy. Decisions about employing technology in distance caregiving inevitably involve weighing, balancing, and rationalizing these concerns, shaping precisely how caregivers’ perceived the deployment and introduction of different kinds of technological interventions. The ethical importance of fulfilling duties of responsibility and maintaining parents’ safety in care may be due to the unique caregiving needs and challenges faced by this sample of only child distance caregivers. Yet, at the same time, the recognition of the irreplacable value of the human connections that comprise relationship-based care, also led some clear limits to be placed on the instrumental value of technological support. Nevertheless, the participants in our study provided different and unique ways to come to a suitable decision for themselves and their parents at a distance. These findings also highlighted the complexity of this decision-making process and emphasized the need to consider a diversified approach that takes into account the needs of each family. Drawing upon the ethics of care theory and literature in transnational care, the use of studied technologies facilitate and validate care activities at a distance, particularly in the phases of *caring about* and *taking care of*, as well as through the management of elder care. Nevertheless, they remain limited in the hands-on phase of *caregiving.*

## Electronic supplementary material

Below is the link to the electronic supplementary material.

Supplementary Material 1

Supplementary Material 2

## Data Availability

Not all data have been granted permission by our participants to be used beyond the scope of this study. Some may be shared on request within a time limit.

## Footnotes

1 Due to the varying terms coined to illustrate the physical barriers present in caregiving as indicating the distance, length of time or costs to travel, this paper will use the terms “transnational-” or “distance-caregivers” interchangeably.

2 For the purpose of this study, we use the term “only children” or “only child transnational *or* distance caregivers” interchangeably to denote those that are over 18, do not have any siblings, with elder care responsibilities to their parents, and have migrated transnationally.
