# Supplementary material for: Distance caregiving using smart home technologies: balancing ethical priorities in family decision-making by only children
Source: BMC Med Ethics. 2025 Jul 3;26:74. doi: 10.1186/s12910-025-01210-8 (PMC12225457; doi:10.1186/s12910-025-01210-8)
Supplement: Supplementary file 1 — Supplementary Material 1 [file 12910_2025_1210_MOESM1_ESM.docx]

Supplemental file 1: Selection of code names and example quotations included (some also in text)

| **Theme in text:** | **Code name:** | **Technology:** | **Example quotation** |
| --- | --- | --- | --- |
| Prioritizing care responsibility to maximize parents’ physical well-being | ***Efficiency in emergency*** | Smart wearables | *“I'm very happy that both of them continue to wear their Apple watches, because the Apple watch is...it has that functionality, both of my parents know how to...send a message or make a phone call using their Apple watch and the Apple watch, as you know, it has the sudden fall technology built into it. So if there is a fall, the phone says, do you need me to call SOS, and if you don't respond in a certain amount of time, it automatically calls, and both of their watches have that functionality. I feel good about that already, you know?”* |
| Prioritizing care responsibility to maximize parents’ physical well-being | ***There is no ethics between family*** | All technologies | *“I think this..could be the purpose of this product to protect privacy, but I think if it is just between relatives, it is not necessary”* |
| Respect for autonomy | ***Delegation of responsibility /respecting autonomy*** | All technologies | *“Yeah, well. If..if..if that is the case, then I'm gonna respect their choice, I mean like, you can't, I mean, even though I buy them the Apple Watch and they don't want to wear it, I...I wouldn't know right? (laughs) I can't force them. So of course, I would first...like consult their option and just ask...if they would love to...kind of wear a watch, or even installing a camera or something. But...it's their will”* |
| Respect for dignity | ***Parents are not pets to surveil*** | All technologies | *“If I have installed this thing and I don't tell them, then I think...this is a very disrespectful behaviour. Because no matter what age someone is, they still...have their own will. They are not some puppet that could be controlled at anytime by someone. You have to clear...if this were to happen to me, I am older and my children...using their words, for my health, to monitor my health, when I am unaware have installed these things...but if I found out later, I would be very angry. I would feel... "Do you not see me as a human being?" Unless I am an animal? I don't have my own opinion/will. It is like surveillance. That's how I would feel.”* |
| Intrinsic need for familial companionship | ***Tech improve caregiving relationship?***  ***Limits of technology to care at a distance***  ***Replacing human care/eliminating care profession*** | All technologies | *“Hm. I mean obviously they, they would probably find it very weird (laughs) if I...show up with that little guy. You know, it sends a signal, obviously. It sends a signal, because you are trying to replace the caregiving...that I, as their son want to give them. I mean, it's more like a substitution. Or it could be seen as a substitution, which could probably negatively impact the relationship. […] Yeah "here you go, I don't want to care about you anymore, here is a robot, that's it."* |

Map #1 of a small selection of codes and their relation to the overall code map:

Theme:

Protection of privacy

Theme:

Respect for dignity

Theme:

Respect for autonomy

Theme:

Prioritizing care responsibility to maximize parents’ physical well-being

***Parents decide who to share with, not children***

***Repugnant against cameras***

***Sensitive vs. non-sensitive data***

***Safety vs. Privacy***

***Dignity***

***Parents are not pets to surveil.***

***Choice to turn on/off***

***If OP accepts it***

***Delegation of responsibility***

***Privacy vs. increasing care needs***

***There is no ethics between family***

***Efficiency in emergency***

Map #2 of a small selection of codes and their relation to the overall code map:

***Replacing human care / eliminating care profession***

***Humanoid robot / deception?***

***For intimate tasks***

***Provides IADL/ADL support***

***Need warm/ emotional care***

***Provides social / entertainment for OP***

***Limits of technology for care at distance***

***Tech improve caregiving relationship?***

Theme:

No technology should be involved in caregiving

Theme:

Increasing health needs call for more support

Theme:

Supplementing emotional connections

Theme:

Intrinsic need for familial companionship
