# Supplementary material for: Distance caregiving using smart home technologies: balancing ethical priorities in family decision-making by only children
Source: BMC Med Ethics. 2025 Jul 3;26:74. doi: 10.1186/s12910-025-01210-8 (PMC12225457; doi:10.1186/s12910-025-01210-8)
Supplement: Supplementary file 2 — Supplementary Material 2 [file 12910_2025_1210_MOESM2_ESM.docx]

# **Interview Guide**

| **Basic Questions** |
| --- |
| Ice-breakers   - Could you tell me a little about your household and your older parent(s)?   - Where are they located and how your relationship is?   - Why good/not good? |
| Questions on caregiving   - How often do you have contact with your older parent(s)? (visit/calls etc.) - Towards your parents living in [NAME OF THE COUNTRY], what are the things that you do to ensure their well-being? (Probe – if nothing is done since parents are very healthy, what do you expect that you will have to do to ensure their well-being) - What are your caregiving duties/responsibilities? - What do you think about your caregiving duties? - What would you like to change about your caregiving roles/duties, if you could change them? - Does being at a distance and providing such change the perception of your responsibility towards your parents? [Explain if question not clear to the participant, i.e. does it care make your responsibilities smaller or bigger or easier or more challenging] Could you explain. - What are some reasons one would take care of older parents? |

| **Core Survey questions** [technology is presented to all participants in pictures or videos] |
| --- |
| 1. **Emergency alarms - red cross button:** you may have heard about these bracelets, seen them, maybe on your parent(s)?    1. What is your opinion about these red-cross buttons?    2. How would you feel about your older parent(s) who is in need of care using such alarm button?       1. Probe: why they think it is good or bad for older persons to use such alarms?       2. How does it help the caregiver specifically? |
| 1. **Sensors in a watch:** what if such sensors could be integrated into a watch (such as the ones I just showed you)    1. What is your general opinion about these watches?    2. What benefits/problems do you see for you when older [mother/father] in using such a smart-watch or a smartphone?   probe: if the benefits and problems above were mentioned for Older person only, ask What benefits for them as caregivers they see?   1. **What if such sensors were also monitoring and recording their health-related data**, for example, heart rate, blood sugar, sleeping schedule, physical activities?    1. What is your general feelings about sensors monitoring health data?    2. If such data were being generated for the older person you are caring for, which third-person do you think should absolutely not see the data?       1. Probe: Who could or should see such data? |
| 1. **Monitoring sensors installed inside of the house** (Imagine that you as the caregiver: could install sensors outside the house of your person in need of care to monitor their safety or to detect falls:    1. Would you consider installing this for your older parent(s)? – Why (not)? [Informal caregiver only because only informal caregivers probably have the power to do this]    2. What benefits/problem do you see with installing such sensors for your older parent?       1. Probe (if problem stated in relation to dignity, privacy, etc.) why would this be a problem? |
| 1. **What if these sensors inside the house were also connected to a camera that generates video-footage?**    1. How would you feel about videos if your person in need of care were not recognizable in it? [e.g. graphical view is shown and not your real image]    2. Would you agree to use such technology if they served a caregiving purpose? Why or why not?    3. What are your concerns with such video technology to monitor your patient or parent? |
| 1. **Now we have discussed all the monitoring technologies. Now I will show you all of them side by side on a sheet of paper.**     1. Which technology would you most likely organize to use for your person in need for caregiving purposes? Why?    2. Which technology would you definitely not use? Why not?   ------Probe about privacy   - 1. Would you recommend such technologies without telling your person in need that they also have a monitoring function, or telling them that they are installed?  1. **All these technologies collect data about their older end-users. I would now like to ask you a few questions about the control of this data.**     1. What do you think about sensors that analyze user’s health data?    2. What do you think about technologies that record and share data about their falls and the location of their fall? [Here we want to know what they actually find useful for them with respect to caregiving] 2. **Ethics (ask if ethical problems have not yet arisen)**    1. In general, what ethical problems do you see with such technologies?   ----Probe for privacy if not yet mentioned   1. **Social relationships**    1. How would your parents react if you would provide her with these monitoring technologies?    2. To what extent would their reactions influence your decision or choice to provide them with these technologies?    3. Do you think that your decision in regards to these technologies would influence the relationship with this older parent? |
| 1. **I would now like to show you a robot**, which is an example of smart technology that could serve as a companion for older persons **[VIDEO OF TELEPRESENCE ROBOT]**    1. What is your opinion on this robot?    2. What problems/concerns/issues do you see with using robots for caring purposes?       1. Why and for whom are these problems concerning?    3. How would you feel about providing each of these for your parents in need of care?    4. What would be your motivation in buying them / one for your older mom/data?    5. Do you think that your recommendation in regards to these technologies would influence the relationship you have with your older patient/older person that you care for?    6. What ethical problems do you see with this robot? |
| 1. **All the technologies that we have discussed so far could be used to make caregiving easier.**    1. Imagine now you are 80 years old and your friend or family gifts you each of these technologies. What would be your opinion on this, and how (would) your opinion differ between these technologies?    2. What role would SHHTs play in your responsibility towards your parents?    3. What would be a problem with providing care from a distance that you foresee, which would prompt the use of smart home technologies?       1. Probe: Why these technologies in particular?    4. How would you evaluate a technology to deem it acceptable, before you recommend it or install it in your parent(s)’ home?       1. Probe: What would be your criteria?    5. What would make you question and stop using a type of smart home technology?       1. Probe: Could you give any examples?    6. What feedback from your parent(s) regarding their use of a smart home technology would make you stop or advise her/him to stop using it?    7. What caregiving challenge would SHHT not be able to solve at a distance?    8. Under what conditions do you think we, as a society, could or should use such solutions to care for older persons?    9. Under what conditions do you think it would be wrong to use such technologies to care for the elderly?    10. What would be your opinion on using smart technologies such as sensors, video cameras, and robots to make caregiving responsibilities easier for family caregivers?    11. Is there a point where some technologies are more acceptable than others when used for caregiving purposes? Why?    12. What general concerns (financial, social, ethical, or otherwise) would you have about smart technologies in general? |
| 1. **If you could choose for a technology to be developed in the future, which capabilities of a robot or technology would be particularly useful/interesting for caregiving for the elderly?** (i.e. To help with cooking, in the garden, as a recording device (to read a book to the grandchildren), as a conversation partner, for shopping for clothes, etc.). |
| 1. **How could the acceptance of smart home technologies be improved in the future?** 2. **Should acceptance be improved?**    1. If yes, on what grounds?    2. If no, what would be your reasons? |
| Is there anything you would like to add that we have not discussed with you yet?  THANK YOU! |
